# Assessment of the confidence interval in the multivariable normal tissue complication probability model for predicting radiation-induced liver disease in primary liver cancer

**DOI:** 10.1093/jrr/rrab011

**Published:** 2021-04-24

**Authors:** Anussara Prayongrat, Natchalee Srimaneekarn, Sira Sriswasdi, Yoichi M Ito, Norio Katoh, Masaya Tamura, Yasuhiro Dekura, Chie Toramatsu, Chonlakiet Khorprasert, Napapat Amornwichet, Petch Alisanant, Yuichi Hirata, Anthony Hayter, Hiroki Shirato, Shinichi Shimizu, Keiji Kobashi

**Affiliations:** 1 Division of Radiation Oncology, Department of Radiology, Faculty of Medicine, Chulalongkorn University, Bangkok, Thailand; 2 Department of Anatomy, Faculty of Dentistry, Mahidol University, Bangkok, Thailand; 3 Research Affairs, Faculty of Medicine, Chulalongkorn University, Bangkok, Thailand; 4 Computational Molecular Biology Group, Faculty of Medicine, Chulalongkorn University, Bangkok, Thailand; 5 Biostatistics Division, Clinical Research and Medical Innovation Center, Hokkaido University Hospital, Sapporo, Japan; 6 Department of Radiation Oncology, Faculty of Medicine, Hokkaido University, Sapporo, Japan; 7 Department of Medical Physics, Hokkaido University Hospital, Sapporo, Japan; 8 Department of Radiation Oncology, Graduate School of Medicine, Hokkaido University, Sapporo, Japan; 9 Department of Radiation Oncology, Tokyo Women’s Medical University, Tokyo, Japan; 10 Central Institute of Isotope Science, Hokkaido University, Sapporo, Japan; 11 Department of Business Information and Analytics, University of Denver, CO, USA; 12 Global Center for Biomedical Science and Engineering, Faculty of Medicine, Hokkaido University, Sapporo, Hokkaido, Japan; 13 Department of Proton Beam Therapy, Faculty of Medicine, Hokkaido University, Sapporo, Hokkaido, Japan; 14 Department of Radiation Medical Science and Engineering, Faculty of Medicine, Hokkaido University Graduate School of Medicine, Sapporo, Japan

**Keywords:** normal tissue complication probability, radiation-induced liver disease, multivariable, confidence interval, prediction model

## Abstract

We developed a confidence interval-(CI) assessing model in multivariable normal tissue complication probability (NTCP) modeling for predicting radiation-induced liver disease (RILD) in primary liver cancer patients using clinical and dosimetric data. Both the mean NTCP and difference in the mean NTCP (ΔNTCP) between two treatment plans of different radiotherapy modalities were further evaluated and their CIs were assessed. Clinical data were retrospectively reviewed in 322 patients with hepatocellular carcinoma (*n* = 215) and intrahepatic cholangiocarcinoma (*n* = 107) treated with photon therapy. Dose–volume histograms of normal liver were reduced to mean liver dose (MLD) based on the fraction size-adjusted equivalent uniform dose. The most predictive variables were used to build the model based on multivariable logistic regression analysis with bootstrapping. Internal validation was performed using the cross-validation leave-one-out method. Both the mean NTCP and the mean ΔNTCP with 95% CIs were calculated from computationally generated multivariate random sets of NTCP model parameters using variance–covariance matrix information. RILD occurred in 108/322 patients (33.5%). The NTCP model with three clinical and one dosimetric parameter (tumor type, Child–Pugh class, hepatitis infection status and MLD) was most predictive, with an area under the receiver operative characteristics curve (AUC) of 0.79 (95% CI 0.74–0.84). In eight clinical subgroups based on the three clinical parameters, both the mean NTCP and the mean ΔNTCP with 95% CIs were able to be estimated computationally. The multivariable NTCP model with the assessment of 95% CIs has potential to improve the reliability of the NTCP model-based approach to select the appropriate radiotherapy modality for each patient.

## INTRODUCTION

Radiotherapy (RT) has been one of the backbone treatments in primary liver cancers including hepatocellular carcinoma (HCC) and intrahepatic cholangiocarcinoma (ICC). However, radiation-induced liver disease (RILD) remains a dose-limiting complication of traditional liver RT and can lead to deterioration of liver function followed by liver failure and death [1]. Currently, standard treatment for RILD has not yet been established, and management is limited to symptomatic and supportive care.

The relationship between dose irradiated to the liver and the incidence of RILD has been investigated for years. According to the Quantitative Analyses of Normal Tissue Effects in the Clinic (QUANTEC) guideline, a mean dose to the normal liver of ≤30–32 Gy is recommended to avoid RILD [2]. In addition, there have been normal tissue complication probability (NTCP) models for predicting risk of RILD based on dose–volume statistics and mathematical models, among which the most common is the Lyman–Kutcher–Burman (LKB) model [3–7]. A number of clinical risk factors other than the dosimetric profile were reported for RILD, including male gender, Child–Pugh (CP) classification, viral hepatitis infection, presence of portal vein thrombosis (PVT), and prior and concurrent treatments [3, 5, 7–9]. It was also reported that worsening of liver function not related to irradiation is often difficult to be distinguished from RILD; confounding factors, such as other locoregional treatments, worsening of cirrhosis itself or other hepatotoxic effects, may also be related to the hepatic toxicities [10].

El Naqa *et al*. introduced a multivariable NTCP modeling in 2006 that combines clinical risk factors and dose–volume factors using a logistic regression framework and data mining [11]. The multivariable NTCP model can be a useful tool to estimate risk of toxicity in individual patients with various clinical backgrounds and translate the dosimetric benefit into clinically relevant benefit. Many investigators have been studying multivariable NTCP models for predicting various toxicities, especially in head and neck (HN) cancers [12–16]. However, there is no multivariable NTCP model for prediction of RILD in patients with primary liver cancer.

Recently, the multivariable NTCP model has attracted attention as a clinical decision support strategy since it helps in comparing between two RT modalities and decision-making to choose the most appropriate RT for each patient. Dose reduction from advanced RT techniques, such as proton beam therapy (PBT), can be translated into a reduction in toxicity, represented by an NTCP reduction [17, 18]. Langendijk *et al*. first proposed the approach of using the multivariable NTCP model for selecting patients for PBT in 2013, referred to as the NTCP model-based approach [19, 20]. Briefly, PBT is suitable for a patient whose difference in the mean NTCP (ΔNTCP) is larger than the pre-defined threshold in their approach [21, 22].

The multivariable NTCP model-based approach has currently been under investigation in HN cancer [18, 21]. Even when a multivariable NTCP model fits well with the validation datasets, the uncertainty of the predicted NTCP value and ∆NTCP value remains the most difficult obstacle for use of the model-based approach in clinical practice [23]. Uncertainty in dose delivery and model parameters were associated with model uncertainty, which significantly affected the accuracy of a model-based approach [24]. There has been a previous study assessing the uncertainty or the confidence interval (CI) for the LKB model [7], but not for a multivariable NTCP model for RILD. Underestimation of NTCP and ∆NTCP can lead to the loss of the opportunity to benefit from PBT, whereas overly cautious practice might cause unnecessary use of this high-cost treatment. Therefore, assessing the uncertainties for both multivariable NTCP and ∆NTCP should improve the reliability of the model prediction in the general population.

The purpose of this study is to develop a multivariable NTCP model for RILD in primary liver cancer patients and to propose a CI-assessing model to evaluate the 95% CI of both the mean NTCP and mean ΔNTCP between two radiotherapy modalities.

## MATERIALS AND METHODS

We retrospectively collected the data of primary liver cancer patients who were treated between December 2006 and September 2018. Inclusion criteria included (i) diagnosis of primary liver cancers (HCC and ICC); (ii) ECOG 0–2; (iii) RT completion; (iv) available three-dimensional (3D) dosimetric parameters; and (v) available follow-up data for tumor and liver toxicity, with at least 4 months of follow-up for non-toxicity patients. We excluded patients who had progressive disease during a 4-month follow-up. All patients were treated with either 3D conformal radiotherapy (3D-CRT), intensity-modulated radiotherapy (IMRT), volumetric arc therapy or stereotactic body radiotherapy (SBRT). A planning contrast-enhanced computed tomography (CT) scan was acquired and used for target delineation, with a 1.0 cm margin expanded to account for subclinical disease, set-up uncertainty and respiratory motion. Treatment verification by cone-beam CT was performed at the first fraction and then weekly. Adjuvant or concurrent fluorouracil-based chemotherapy was administered in ICC patients with locally advanced disease. The study was approved by the local institutional review board (IRB no. 602/60).

RILD is classified as ‘classic’ and ‘non-classic’ RILD. The clinical manifestation of classic RILD includes anicteric hepatomegaly, ascites and elevated alkaline phosphatase more than twice the upper limit of normal (ULN). In contrast, non-classic RILD involves elevated liver transaminases [aspartate aminotransferase (AST) and alanine aminotransferase (ALT)] more than 5× ULN within 3 months after therapy, or liver function deterioration measured by a decline in CP score by ≥2, with the absence of classic RILD [25]. The non-classic form tends to develop in patients with underlying poor hepatic function such as viral hepatitis infection status or cirrhosis [1]. In the current study, classic RILD was the endpoint for CP-A and negative viral hepatitis infection status patients. Non-classic RILD was scored for CP-B or CP-C, or positive viral hepatitis infection status patients.

### Data extraction of clinical and dosimetric parameters

Baseline patient characteristics including age, gender, number of tumors, tumor size, presence of PVT, CP score, viral hepatitis B and C (HBV and HCV) infection status and previous treatments were reviewed. Blood tests and imaging were assessed at baseline and every 1–3 months after completing RT for evaluation of treatment-related toxicities and disease progression.

Dose–volume histograms (DVHs) of all patients were obtained from the Eclipse planning system version 8.6 (Varian Medical Systems, Palo Alto, CA, USA). To simplify the whole DVH into a single measurement and simultaneously account for organ architecture, normal liver DVH was reduced to equivalent uniform dose (EUD). The mean liver dose (MLD) was a cumulative result of fraction size-adjusted EUD in each dose bin associated with the partial volume associated with that dose bin (Supplementary Data 1).

### Statistical analysis

Clinical and dosimetric parameters between patients with or without RILD were compared using a χ^2^ test or *t*-test. Overall survival (OS) was estimated from the date of start of RT to the date of death or last follow-up using the Kaplan–Meier method, and compared between groups using the log-rank test.

A univariate analysis was initially performed for all candidate predictors potentially to be used in the multivariable NTCP model. Spearman’s rank correlation (*R*) was tested to avoid multicollinearity issues. If the correlation between multiple variables was >0.65, only one variable was selected as representative and included in the subsequent multivariate analysis. Further, multivariate logistic regression analysis with bootstrapping technique and forward variable selection was performed. Regression coefficients, the odds ratio (OR) and the 95% CI were obtained for each variable as well as their variance–covariance matrix. For an individual patient, the risk of RILD (NTCP value) can be estimated using the following equation:

$\mathrm{NTCP}$ = $\frac{1}{1+{e}^{-S(x)}}$, in which.

$S(x)$ = ${\beta}_0+{\beta}_i{x}_{i.}$

Where ${x}_i\ (1\le i\le n)$ is the prognostic variable, ${\beta}_0$ is a constant value and ${\beta}_i\ (1\le i\le n)$ represented the regression coefficients for the *i*th covariate ${x}_i$. According to TRIPOD guidelines, splitting data into training and validation sets is inefficient for internal validation of a predictive logistic regression model [26]. Therefore, we decided to use all data for model generation and perform cross-validation leave-one-out method (LOO-CV) which is suitable for a limited amount of data.

Since the first objective of this study is to develop the multivariable NTCP model for predicting RILD, we assessed the model performance by various measures including discriminative ability using the area under the receiver operative characteristics curve (AUC), agreement between predicted and observed outcomes using the calibration plot, Nagelkerke’s *R*^2^ and scaled Brier score. Hosmer–Lemeshow (HL) goodness-of-fit test was further used to assess calibration between the observed and predicted outcomes. Statistical analyses were conducted in STATA LP (version 13.1, StataCorp, College Station, TX, USA) and R version 3.4.2 (R Core Team).

### Estimation of NTCP reduction (∆NTCP)

With model regression coefficients (β), the ∆NTCP between two treatment plans, i.e. X-ray therapy (XRT) versus PBT in this study, was estimated. To demonstrate ∆NTCP for all possible dose differences between XRT versus PBT plans, the ∆NTCP values were calculated at every MLD level for each treatment plan (0 to 80 Gy with 1 Gy intervals). The desired ∆NTCP values, e.g. 10% according to the ∆NTCP threshold for PBT in the Netherlands [19], was illustrated on a ∆NTCP map where each coordinate represented the dose at the PBT plan resulting in 10% ∆NTCP in comparison with the XRT plan.

### Assessment of uncertainty in the NTCP model

Model uncertainty was assessed using a probability distribution of the model parameters by simulation from a multivariate normal distribution (1000 iterations). Based on the central limit theorem for the multivariate statistics, if a collection of random vectors (${x}_i$) was independently sampled from a population with mean vector (μ) and variance–covariance matrix (Σ), and these random vectors were identically distributed, then the sample mean vector ($\overline{x}$) approximated the mean vector (μ). According to the multidimensional central limit theorem, when scaled, summation of these vectors converged to a multivariate normal distribution [27]. In the current study, we generated 1000 sets of random coefficients (${\beta}_r)$assuming a multivariate normal distribution of model parameters with μ equal to the regression coefficients (${\beta}_0\ \mathrm{and}\ {\beta}_i$) and Σ their variance–covariance matrix. Each set of model coefficients was then used for NTCP calculation where the mean and 95% CI were obtained. Afterwards, the ∆NTCP between XRT and PBT was estimated for the mean and 95% CI.

## RESULTS

### Distribution of patients

The majority of the patients were male (74.2%) and of CP-A classification (73.3%). Half of the patients had a positive viral hepatitis infection status. The median prescription dose was 45 Gy per daily fraction. The pre-treatment characteristics are listed in Table 1. The incidence of RILD in patients overall was 33.5% (108/322). With the median follow-up time of 8.6 months, median OS for patients overall was 13.4 months (95% CI 11.7–16.7 months): 17.9 months (95% CI 16.7–22.7 months) versus 5.4 months (95% CI 4.2–6.5 months) in patients with non-RILD versus RILD, respectively (*P* < 0.001).

**Table 1:** Pre-treatment characteristics

	All (*n* = 322)	RILD (*n* = 108)	Non-RILD (*n* = 214)	*P* value
Mean (SD) age (years)	60.5 (12.2)	59.5 (11.5)	61.1 (12.5)	0.29
Gender				0.07
Female	83 (25.8)	21 (19.4)	62 (29)	
Male	239 (74.2)	87 (80.6)	152 (71)	
Primary disease				<0.001
Hepatocellular carcinoma	215 (66.8)	99 (91.7)	116 (54.2)	
Intrahepatic cholangiocarcinoma	107 (33.2)	9 (8.3)	98 (45.8)	
Presence of portal vein thrombosis	166 (51.6)	80 (74.1)	86 (40.2)	<0.001
T classification				0.01
Early T stage (T2 or less)	98 (30.4)	22 (20.4)	76 (35.5)	
Late T stage (T3 or more)	224 (69.6)	86 (79.6)	138 (64.5)	
N classification				0.15
No lymph node involvement	247 (76.7)	88 (81.5)	159 (74.3)	
Positive lymph node involvement	75 (23.3)	20 (18.5)	55 (25.7)	
Child–Pugh classification				<0.001
A	236 (73.3)	65 (60.2)	171 (79.9)	
B or C	86 (26.7)	43 (39.8)	43 (20.1)	
Viral hepatitis B or C infection	168 (52.2)	84 (77.8)	84 (39.3)	<0.001
Previous treatments				
Surgery	83 (25.8)	12 (11.1)	71 (33.2)	<0.001
Transarterial chemoembolization	139 (43.2)	56 (51.9)	83 (38.8)	0.03
Chemotherapy	46 (14.3)	10 (9.3)	36 (16.8)	0.07
Radiation technique				0.99
3D-CRT	127 (39.4)	43 (39.8)	84 (39.3)	
IMRT/VMAT	137 (42.6)	46 (42.6)	91 (42.5)	
SBRT	58 (18)	19 (17.6)	39 (18.2)	
Median (IQR) total dose (Gy)	45 (30–50)	30 (30–45)	45 (30–50)	<0.001
Median (IQR) number of fractions	10 (10–25)	10 (7–10)	15 (10–25)	<0.001
Median (IQR) dose per fraction (Gy)	3 (1.8–4)	3 (3–4)	3 (1.8–3)	<0.001
Mean (SD) normal liver volume	1235.6 (530.7)	1224.4 (609.7)	1241.0 (489.4)	0.800
Mean (SD) liver dose (Gy)	17.6 (7.8)	19.0 (8.8)	16.9 (7.2)	0.023
Mean (SD) effective volume (ml)	0.4 (0.5)	0.5 (0.8)	0.3 (0.2)	0.067
Mean (SD) D_10%_ (Gy)	42.5 (18.2)	45.0 (20.7)	41.3 (16.7)	0.086
Mean (SD) D_20%_ (Gy)	29.8 (13.6)	31.2 (13.9)	29.1 (13.4)	0.174
Mean (SD) D_30%_ (Gy)	22.2 (12.0)	23.1 (11.7)	21.7 (12.2)	0.328
Mean (SD) D_40%_ (Gy)	16.9 (10.4)	17.7 (10.1)	16.5 (10.6)	0.350
Mean (SD) D_50%_ (Gy)	12.9 (8.9)	13.7 (9.1)	12.5 (8.9)	0.267

Abbreviations: RILD = radiation-induced liver disease, 3D-CRT = three-dimensional conformal radiotherapy, IMRT = intensity modulated radiotherapy, VMAT = volumetric modulated arc therapy, SBRT = stereotactic body radiotherapy, V_x Gy_ = volume of normal liver receiving x Gy, D_x %_ = dose irradiated to x% volume of normal liver, IQR = interquatile range, SD = standard deviation.

Table 2 and Supplementary Data 2 demonstrate univariate analysis and Spearman correlation test between variables, and all candidate predictors potentially to be used in the multivariable NTCP model, respectively.

**Table 2:** Univariate logistic regression analysis for radiation-induced liver disease

Variables	Odds ratio	95% CI		*P* value
		Lower	Upper	
Age (years)	0.990	0.971	1.009	0.286
Male (versus female)	1.690	0.965	2.960	0.067
Primary disease of HCC (versus ICC)	9.293	4.463	19.351	<0.001
Presence of portal vein thrombosis (versus no)	4.252	2.554	7.080	<0.001
Late T stage (versus early T stage)	2.153	1.248	3.715	0.006
N1 (versus N0)	0.657	0.370	1.167	0.152
Child–Pugh B or C (versus A)	2.631	1.580	4.382	<0.001
Positive viral hepatitis status (versus negative)	2.452	1.518	3.958	<0.001
Previous treatments				
No surgery (versus surgery)	3.972	2.044	7.717	<0.001
TACE (versus no TACE)	1.700	1.066	2.711	0.026
No chemotherapy (versus chemotherapy)	1.982	0.943	4.166	0.071
Radiation technique				
3D-CRT (versus non-3D)	1.024	0.638	1.643	0.922
SBRT (versus non SBRT)	0.958	0.523	1.754	0.889
Normal liver volume	1.000	0.999	1.000	0.801
Mean liver dose (Gy)	1.035	1.005	1.067	0.024
Effective volume (ml)	2.997	1.091	8.234	0.033
D_10%_ (Gy)	1.011	0.998	1.024	0.090
D_20%_ (Gy)	1.012	0.995	1.029	0.174
D_30%_ (Gy)	1.010	0.990	1.029	0.327
D_40%_ (Gy)	1.011	0.988	1.033	0.349
D_50%_ (Gy)	1.015	0.989	1.041	0.267

Abbreviations: OR = odds ratio, 95% CI = 95% confidence interval, ICC = intrahepatic cholangiocarcinoma, HCC = hepatocellular carcinoma, TACE = transarterial chemoembolization, 3D-CRT = three-dimensional conformal radiotherapy, SBRT = stereotactic body radiotherapy, V_x Gy_ = volume of normal liver receiving x Gy, D_x %_ = dose irradiated to x% volume of normal liver.

### NTCP model development

In the multivariate logistic regression analysis with 1000 bootstrapping, the significant variables in the final model included diagnosis of HCC, CP-B or CP-C, positive hepatitis and MLD. The regression coefficients, OR and 95% CI of each selected variable are shown in Table 3. The NTCP value for the individual patient can be calculated using the equation:

**Table 3:** Multivariable logistic regression analysis for radiation-induced liver disease

Variables	ORs	95% CI		*P* value	Regression coefficient	95% CI		Multiplication value
		Lower	Upper			Lower	Upper	
**Diagnosis**								
ICC	Ref				Ref			
HCC	5.64	2.21	12.99	<0.001	1.73	0.80	2.56	0 = no, 1 = yes
**Child–Pugh classification**								
A	Ref				Ref			
B or C	2.36	1.30	4.23	0.004	0.86	0.26	1.44	0 = no, 1 = yes
**Viral hepatitis B or C infection**								
Negative	Ref				Ref			
Positive	2.73	1.51	4.72	<0.001	1.00	0.41	1.55	0 = no, 1 = yes
**Mean liver dose**	1.05	1.01	1.09	0.007	0.05	0.01	0.08	Dose in Gy
**Constant**	0.02	0.08	0.09	<0.001	−3.70	-4.83	-2.42	

Abbreviations: OR = odds ratio, 95% CI = 95% confidence interval, ICC = intrahepatic cholangiocarcinoma, HCC = hepatocellular carcinoma; Ref = reference.

$\mathrm{NTCP}$ = $\frac{1}{1+{e}^{-S(x)}},$ in which

$S(x)$ = −3.70 + (diagnosis of HCC × 1.73) + (CP-B or C × 0.86) + (viral hepatitis infection status × 1.00) + (MLD × 0.05)

The overall model performance was good, indicated by an AUC of 0.79 (95% CI 0.74–0.84), Nagelkerke’s *R*^2^ of 0.31 and scaled Brier score of 0.24. The HL test showed an agreement between predicted and observed outcomes (χ^2^ = 1.54, *P* = 0.67). The model has high accuracy (73.9%), specificity (84.1%) and negative predictive value (78.3%).

**Fig. 1.**
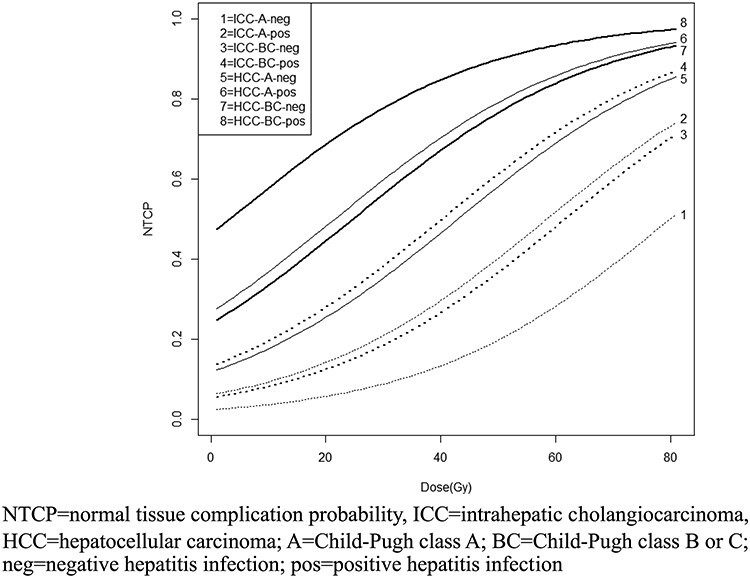
NTCP curves as a function of mean liver dose for eight patient subgroups classified by three significant clinical characteristics.

According to three clinical parameters (diagnosis, CP class and hepatitis infection status), patients were classified into eight possible subgroups. For each patient subgroup, MLD was the only factor that contributed to an NTCP curve (Fig. 1).

### Estimation of the mean and 95% CI for NTCP and ∆NTCP

**Fig. 2.**
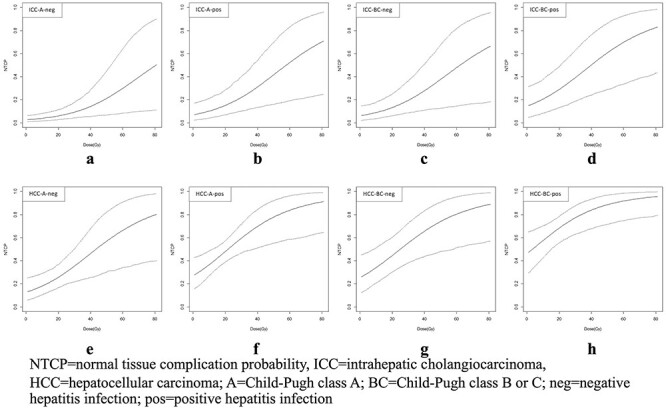
Schema of assessing model uncertainties by simulation from a multivariate normal distribution of model coefficients with a variance–covariance matrix.

**Fig. 3.**
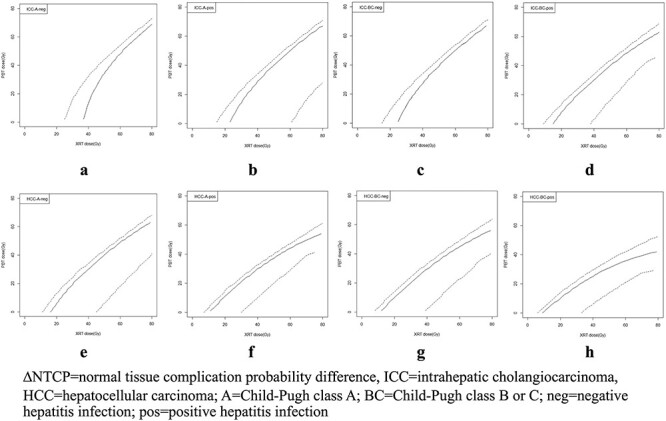
∆NTCP map: iso-10% ∆NTCP contour with the 95% confidence interval for eight patient subgroups classified by three significant clinical characteristics.

With model regression coefficients (${\beta}_0\hbox{--} {\beta}_4$) and their variance–covariance matrix (Supplementary Data 3), a simulation from a multivariate normal distribution resulted in a total of 1000 sets of random coefficients (${\beta}_{r0-}{\beta}_{r4}$) which were used to assess NTCP and ∆NTCP uncertainties (Fig. 2). For each of the eight patient subgroups (fixed ${x}_1\hbox{--} {x}_3$), 1000 NTCP values were calculated at every MLD level (${x}_4$ = 0–80 Gy with 1 Gy intervals). Subsequently, mean NTCP values with 95% CIs for all MLD levels were obtained (Fig. 3). In addition, ∆NTCP between XRT and PBT for each subgroup was given by a function:

**Fig. 4.**
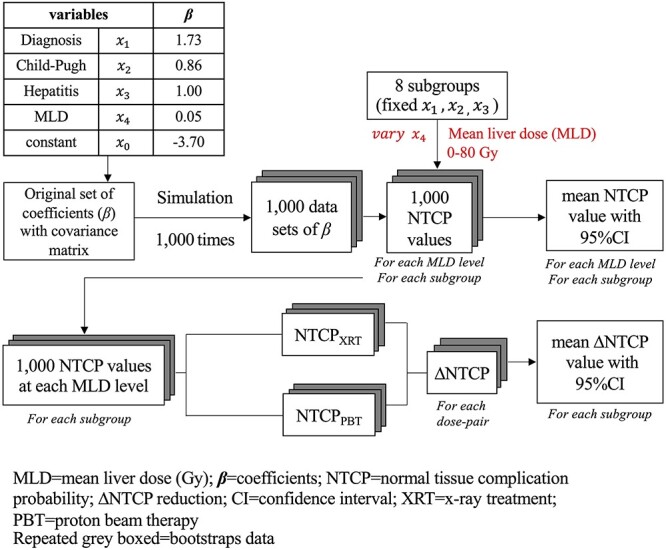
NTCP curves with the 95% confidence interval for eight patient subgroups classified by three significant clinical characteristics.

*f*(XRT, PBT) = NTCP_XRT_ (MLD_XRT_|${\beta}_r$) – NTCP_PBT_ (MLD_PBT_|${\beta}_r$),

where *f* is a function of ∆NTCP between XRT and PBT, and MLD_XRT_ and MLD_PBT_ denote the mean liver dose from the XRT and PBT treatment plan in a certain patient, respectively. ${\beta}_r$ is a set of model regression coefficients from each simulation (iteration).

Therefore, 1000 ∆NTCP values for each pair of MLD_XRT_ and MLD_PBT_ were derived and used to define mean NTCP values with 95% CIs. To illustrate the relationship between ∆NTCP and MLD_XRT_ – MLD_PBT_, we selected a single ∆NTCP value, e.g. 10% according to the ∆NTCP threshold for PBT in the Netherlands [19]. At ∆NTCP = 10%, each MLD_XRT_ and MLD_PBT_ pair represented coordinates on the ∆NTCP map. In our study, not only the mean value but also ∆NTCP = 10% at the lower bound of the 95% CI (95% CI-LB) was plotted (Fig. 4). The line connecting coordinates was called the iso-∆NTCP contour and the area to the right and beneath this contour was considered a PBT-benefit area (∆NTCP >10%).

## DISCUSSION

RILD has persistently been a dose-limiting complication of traditional liver-directed RT, leading to unfavorable treatment outcomes [1, 28]. In this study, we developed the multivariable NTCP model to estimate the risk of RILD. Using multivariate logistic regression analysis, the final model consisted of four significant parameters, namely diagnosis (HCC versus ICC), pre-treatment CP classification (CP-A versus CP-B or C), HBV/HCV infection status (positive versus negative) and MLD. Model performance and validity were good. Furthermore, we first proposed the use of a simulation-based computational method to assess uncertainty of the mean NTCP and the mean ∆NTCP with 95% CIs which would assist decision-making on RT modality selection.

Compared with 9.4–19.1% in previous reports [3–8], the high RILD incidence in this study (33.5%) might be due to greater intrinsic biosusceptibility characteristics and pre-existing liver function abnormality in our patient population. Two-thirds of patients had HCC which is known to be frequently associated with chronic viral hepatitis and liver cirrhosis [4, 6, 9]. Correspondingly, HCC diagnosis was the most significant predictor, with adjusted ORs of 5.64 (95% CI 2.21–12.99, *P* value <0.001). The other possible reason for the high incidence rate of RILD was the use of patient-specific RILD criteria, i.e. classic RILD for CP-A, non-hepatitis patients and non-classic RILD for those with CP-B or C or hepatitis.

The predictive factors for RILD included in our model were mostly in line with previous studies [3, 5, 8, 9, 29, 30]. In the current study, the presence of PVT, late T stage and transcatheter arterial chemoembolization (TACE) were associated with the outcome in univariate analysis, but were eventually excluded from the final model due to an inadequately significant effect. Unlike in an earlier report by Dawson *et al*. [3], chemotherapy was not associated with increased RILD risk in this study (OR = 0.56, *P* = 0.17). A possible explanation could be that, in contrast to concomitant use with twice-daily RT [3], none of our HCC patients received concurrent chemotherapy.

Previous studies reported NTCP models for RILD mainly based on Lyman models. The well-known Michigan study recruited patients with normal liver function but unknown viral hepatitis status, and their treatment regimen was twice-daily RT concurrent with chemotherapy [3, 25]. In Asian studies, CP-B and HBV carrier status were taken into account [5, 8, 29]. Prayongrat *et al*. recently reported the Lyman NTCP models stratified by patient characteristics (CP classification and hepatitis status) and found considerably different NTCP curves between subgroups [7]. In the present study, we first integrated these clinical variables and dose–volume factors into a single model, a so-called multivariable NTCP model.

Predictive models were generally developed from the statistical assumption of a subset of the patient population which resulted in model uncertainty. Bijman *et al*. assessed the uncertainty of a multivariable NTCP model in HN cancers using a probability distribution (mean and CI) of the model coefficients [24], but we could not find any other studies about multivariable NTCP models which referred to uncertainty. The present study reported a new approach to evaluate CIs of NTCP from computationally generated NTCP model parameter estimator sets around the maximum likelihood point. The statistical characteristics of the generated estimator sets coincide with the mean and variance–covariance of the NTCP model parameters. Compared with the Delta method to evaluate variance of NTCP (and its CIs), the approach presented here is advantageous with regards to generalizability since simple computer implementation can be carried out without any complex mathematical formulation and programming, even for NTCP models with many parameters. Due to this feature, the presented approach has good compatibility with multivariable NTCP models as well as evaluation for CIs of ΔNTCP.

It has been known that reducing the dose to the normal liver is the key feature of PBT in liver cancers, and patient selection for PBT based on the multivariable NTCP model-based approach is expected to be useful [19–23]. According to the Dutch Society of Radiotherapy and Oncology consensus, PBT is suitable for patients with ∆NTCP of ≥10% for grade 2 toxicity, in which the mean value or central estimate of ΔNTCP was generally considered for decision-making [31]. On the other hand, Kobashi *et al*. and Prayongrat *et al*. proposed the use of the 95% CI-LB of ΔNTCP as a threshold to be more conservative [7, 32]. According to the definition of CI, there was a 95% probability that the true ΔNTCP value in the population would be larger than the 95% CI-LB whereas there was a 50% probability that the true value would be larger than the central estimate. Therefore, iso-ΔNTCP contours at the 95% CI-LB always served a smaller proton benefit area compared with those at the central estimate. The estimation of the 95% CI for ΔNTCP does at least broaden the range of information for the clinical decision support in patient selection for PBT. The threshold of the CI can be changed from 95% to another percentage if it is more reasonable from any point of view, such as new clinical evidence or the healthcare system in each country.

Other advantages of our model were the comprehensive integration of clinical and dosimetric factors into the model using a relatively large patient dataset, and the simplicity as we stratified patients according to their clinical characteristics into eight subgroups. With a given MLD, one can easily obtain the NTCP with the 95% CI in an individual patient. Also, with MLD from both XRT and PBT plans, a treatment selection can be guided. For example, given MLD = 33 Gy for the XRT plan versus 20 Gy for the PBT plan in patients with HCC, CP-A and negative hepatitis status, the estimated NTCP_33 Gy_ is 39.6% (95% CI 23.4–57.0), NTCP_20 Gy_ is 26.5% (95% CI 17.2–37.9) and ∆NTCP is 13.1% (95% CI 3.0–24.8). Another strong point of this study was the use of fraction size-adjusted dose accounting for a variety of fraction sizes, thus enabling the generalized application of this model among various treatment schedules and fractionations.

A limitation of this study was its retrospective nature. Heterogeneity of disease characteristics and treatment techniques/regimens inevitably affected the predictive power of the model [33]. Another limitation is that various dose fractionation regimens were used for the patients in the dataset. Since calculation of EUDs is a critical step for generating an NTCP model, both dose per fraction and total dose could have affected the probability of liver damage in each bin, depending on the background condition of the liver. Therefore, our model needs external validation prior to clinical application. It is also clear that it is preferable to use this multivariable NTCP model restrictively to calculate ∆NTCP comparing two radiotherapy modalities such as SBRT and PBT for the same patient using the same dose fractionation schedule. It should not be used to compare different dose fractionation schedules or a different total dose even for the same patient. Thirdly, we assumed that the α/β ratio was 2 Gy for RILD. However, this parameter may also contain a large amount of uncertainty from patient to patient. For example, the α/β ratio could be different in cirrhotic liver from normal liver. In future, the model may be more sophisticated by selecting a α/β ratio for cirrhotic liver different from normal liver. Until then, the model needs careful validation and restriction prior to clinical application even for the model-based approach. In addition, the novel predictive markers as well as radiogenomic data have been of greater interest for RILD [34, 35]. These potential biomarkers are capable of being integrated at any time into the multivariable NTCP model for improving prediction ability and reliability.

RILD is a dose-limiting complication in liver cancer. The present study introduced a multivariable NTCP model for RILD and a CI-assessing model to evaluate the 95% CI of both the NTCP and the ΔNTCP between two radiotherapy modalities. In the model-based approach that relied on the ∆NTCP threshold for the same patient, the reliability to select the appropriate radiation modality such as proton therapy would be improved. Further studies should focus on strategies for patient selection for proton therapy and an appropriate ∆NTCP threshold.

## Conflicts of interest

None.

## Funding

This research was supported by grants from the Ministry of Education, Science, Sports, and Culture, Japan (nos 18H02758, 15H04899 and 15H04768).

## Presentation at conference

Poster Discussion session Radiobiological and predictive modelling, and radiomics 1, The European Society of Therapeutic Radiology and Oncology (ESTRO) 2020.

## Supplementary Material

MVA_supplements_07072020_rrab011
