# Supplementary material for: Assessment of the confidence interval in the multivariable normal tissue complication probability model for predicting radiation-induced liver disease in primary liver cancer
Source: J Radiat Res. 2021 Apr 24;62(3):483–93. doi: 10.1093/jrr/rrab011 (PMC8127660; doi:10.1093/jrr/rrab011)
Supplement: MVA_supplements_07072020_rrab011 [file mva_supplements_07072020_rrab011.docx]

**Supplement 1**

To simplify the whole DVH into a single measurement and simultaneously account for organ architecture, normal liver DVH was reduced to equivalent uniform dose (EUD):

$EUD$ = $\left( \sum_{i} v_{i}D_{i}^{\frac{1}{n}} \right)^{n}$

where $v_{i}$is the fractional volume of normal liver that received $D_{i}$Gy in the $i^{th}$ bin and the parameter $n$is a volume effect. Due to large volume effect of parallel organ such as liver, we assumed that $n$=1 resulting in $EUD$ = mean liver dose.

According to the heterogeneity of RT regimens, $D_{i}$ for each dose bin were converted to fraction-size equivalent dose (FED) as follows:

${FED}_{\alpha/\beta}^{fs}$ = ${Nd\left( \frac{1+\frac{d}{\alpha/\beta}}{1+\frac{fs}{\alpha/\beta}} \right)}$

where $f_{s}$ denotes the reference fraction size, $d$is the physical dose per fraction, $N$ is the number of fractions and $\alpha/\beta$ is the ratio of the linear quadratic model parameters for the organ at risk. In this study, the $f_{s}$ =2 Gy and $\alpha/\beta$ ratio=2 Gy were used for calculation.

Mean liver dose (MLD) was a cumulative result of FED in each dose bin associated with partial volume associated in the particular dose bin:

$${MLD = Mean FED}_{\alpha/\beta}^{fs}= \sum_{n=1}^{N_{b}} \left[ {FED}_{\alpha/\beta}^{fs} \right]_{i}v_{i}$$

where $N_{b}$ denotes the total number of dose bins in the differential DVH, $\left[ {FED}_{\alpha/\beta}^{fs} \right]_{i}$ and $v_{i}$ is FED and partial volume in the $i^{th}$ dose bin, respectively.

**Supplement 2** Spearman’s correlation coefficient between variables.

|  | **Gender** | **Diagnosis** | **PVT** | **T stage** | **CP** | **Hepatitis** | **Surgery** | **TACE** | **CMT** | **MLD** | **Veff** | **D10%** | |
| --- | --- | --- | --- | --- | --- | --- | --- | --- | --- | --- | --- | --- | --- |
| **Gender** | 1.000 | .232^**^ | .210^**^ | 0.089 | 0.003 | .175^**^ | -.123^*^ | .112^*^ | -0.064 | -0.004 | -0.066 | -0.029 |  |
| **Diagnosis** | .232^**^ | 1.000 | .569^**^ | .221^**^ | .128^*^ | .512^**^ | -.519^**^ | .562^**^ | -.353^**^ | -0.039 | -0.090 | .121^*^ |  |
| **PVT** | .210^**^ | .569^**^ | 1.000 | .426^**^ | 0.080 | .316^**^ | -.423^**^ | .305^**^ | -.190^**^ | .137^*^ | .110^*^ | -0.033 |  |
| **T stage** | 0.089 | .221^**^ | .426^**^ | 1.000 | 0.079 | 0.042 | -.135^*^ | 0.045 | -0.077 | 0.024 | .139^*^ | -0.018 |  |
| **CP** | 0.003 | .128^*^ | 0.080 | 0.079 | 1.000 | .156^**^ | -.211^**^ | 0.055 | -0.066 | -.113^*^ | 0.025 | .119^*^ |  |
| **Hepatitis** | .175^**^ | .512^**^ | .316^**^ | 0.042 | .156^**^ | 1.000 | -.260^**^ | .345^**^ | -.284^**^ | 0.007 | -0.061 | .111^*^ |  |
| **Surgery** | -.123^*^ | -.519^**^ | -.423^**^ | -.135^*^ | -.211^**^ | -.260^**^ | 1.000 | -.284^**^ | .226^**^ | -0.056 | 0.029 | -0.038 |  |
| **TACE** | .112^*^ | .562^**^ | .305^**^ | 0.045 | 0.055 | .345^**^ | -.284^**^ | 1.000 | -.248^**^ | -.134^*^ | -.302^**^ | 0.062 |  |
| **CMT** | -0.064 | -.353^**^ | -.190^**^ | -0.077 | -0.066 | -.284^**^ | .226^**^ | -.248^**^ | 1.000 | 0.028 | 0.091 | -0.066 |  |
| **MLD** | -0.004 | -0.039 | .137^*^ | 0.024 | -.113^*^ | 0.007 | -0.056 | -.134^*^ | 0.028 | 1.000 | .688^**^ | -.133^*^ |  |
| **Veff** | -0.066 | -0.090 | .110^*^ | .139^*^ | 0.025 | -0.061 | 0.029 | -.302^**^ | 0.091 | .688^**^ | 1.000 | -0.106 |  |
| **D10%** | -0.029 | .121^*^ | -0.033 | -0.018 | .119^*^ | .111^*^ | -0.038 | 0.062 | -0.066 | -.133^*^ | -0.106 | 1.000 |  |

** Correlation is significant at the 0.01 level (2-tailed).

* Correlation is significant at the 0.05 level (2-tailed).

*Abbreviation: PVT=portal vein thrombosis; CP=Child-Pugh classification; TACE=transarterial chemoembolization; CMT= chemotherapy; MLD=mean liver dose; Veff=effective volume; D10%=dose irradiated to 10% volume of normal liver*

**Supplement 3**

Variance-covariance matrix of model coefficients were obtained.

|  | **Diagnosis** | **Child-Pugh** | **Hepatitis** | **Mean liver dose** | **constant** |
| --- | --- | --- | --- | --- | --- |
| **Diagnosis** | 0.1668 | 0.0012 | -0.0400 | 0.0006 | -0.1270 |
| **Child-Pugh** | 0.0012 | 0.0870 | -0.0051 | 0.0006 | -0.0355 |
| **Hepatitis** | -0.0400 | -0.0051 | 0.0926 | 0.0000 | -0.0271 |
| **Mean liver dose** | 0.0006 | 0.0006 | 0.0000 | 0.0003 | -0.0062 |
| **constant** | -0.1270 | -0.0355 | -0.0271 | -0.0062 | 0.2665 |
